# A genotypic method for determining HIV-2 coreceptor usage enables epidemiological studies and clinical decision support

**DOI:** 10.1186/s12977-016-0320-7

**Published:** 2016-12-20

**Authors:** Matthias Döring, Pedro Borrego, Joachim Büch, Andreia Martins, Georg Friedrich, Ricardo Jorge Camacho, Josef Eberle, Rolf Kaiser, Thomas Lengauer, Nuno Taveira, Nico Pfeifer

**Affiliations:** 1 Department for Computational Biology and Applied Algorithmics, Max Planck Institute for Informatics, Saarland Informatics Campus, Campus E 1 4, 66123 Saarbrücken, Germany; 2 Research Institute for Medicines (iMed.ULisboa), Faculty of Pharmacy, University of Lisbon, Av. Professor Gama Pinto, 1649-003 Lisbon, Portugal; 3 Centro de Administração e Políticas Públicas (CAPP), Instituto Superior de Ciências Sociais e Políticas (ISCSP), University of Lisbon, Rua Almerindo Lessa, 1300-663 Lisbon, Portugal; 4 Rega Institute for Medical Research, Clinical and Epidemiological Virology, Department of Microbiology and Immunology, KU Leuven-University of Leuven, Minderbroedersstraat 10, 3000 Louvain, Belgium; 5 Department of Virology, Max von Pettenkofer-Institut, Ludwig-Maximilians-University, Pettenkoferstraße 9a, 80336 Munich, Germany; 6 Institute for Virology, University of Cologne, Fürst-Pückler-Str. 56, 50935 Cologne, Germany; 7 Instituto Superior de Ciências da Saúde Egas Moniz (ISCSEM), Campus Universitário, Quinta da Granja, Monte de Caparica, 2829-511 Caparica, Portugal

**Keywords:** Human immunodeficiency virus type 2, HIV-2, Coreceptor, Chemokine receptor, Prediction, Statistical learning, V3, V1, V2, Coreceptor antagonists

## Abstract

**Background:**

CCR5-coreceptor antagonists can be used for treating HIV-2 infected individuals. Before initiating treatment with coreceptor antagonists, viral coreceptor usage should be determined to ensure that the virus can use only the CCR5 coreceptor (R5) and cannot evade the drug by using the CXCR4 coreceptor (X4-capable). However, until now, no online tool for the genotypic identification of HIV-2 coreceptor usage had been available. Furthermore, there is a lack of knowledge on the determinants of HIV-2 coreceptor usage. Therefore, we developed a data-driven web service for the prediction of HIV-2 coreceptor usage from the V3 loop of the HIV-2 glycoprotein and used the tool to identify novel discriminatory features of X4-capable variants.

**Results:**

Using 10 runs of tenfold cross validation, we selected a linear support vector machine (SVM) as the model for geno2pheno[coreceptor-hiv2], because it outperformed the other SVMs with an area under the ROC curve (AUC) of 0.95. We found that SVMs were highly accurate in identifying HIV-2 coreceptor usage, attaining sensitivities of 73.5% and specificities of 96% during tenfold nested cross validation. The predictive performance of SVMs was not significantly different (p value 0.37) from an existing rules-based approach. Moreover, geno2pheno[coreceptor-hiv2] achieved a predictive accuracy of 100% and outperformed the existing approach on an independent data set containing nine new isolates with corresponding phenotypic measurements of coreceptor usage. geno2pheno[coreceptor-hiv2] could not only reproduce the established markers of CXCR4-usage, but also revealed novel markers: the substitutions 27K, 15G, and 8S were significantly predictive of CXCR4 usage. Furthermore, SVMs trained on the amino-acid sequences of the V1 and V2 loops were also quite accurate in predicting coreceptor usage (AUCs of 0.84 and 0.65, respectively).

**Conclusions:**

In this study, we developed geno2pheno[coreceptor-hiv2], the first online tool for the prediction of HIV-2 coreceptor usage from the V3 loop. Using our method, we identified novel amino-acid markers of X4-capable variants in the V3 loop and found that HIV-2 coreceptor usage is also influenced by the V1/V2 region. The tool can aid clinicians in deciding whether coreceptor antagonists such as maraviroc are a treatment option and enables epidemiological studies investigating HIV-2 coreceptor usage. geno2pheno[coreceptor-hiv2] is freely available at http://coreceptor-hiv2.geno2pheno.org.

**Electronic supplementary material:**

The online version of this article (doi:10.1186/s12977-016-0320-7) contains supplementary material, which is available to authorized users.

## Background

Human immunodeficiency virus type 2 (HIV-2) is prevalent in Western Africa and specific European countries, such as France and Portugal [1]. In comparison to HIV-1, HIV-2 exhibits a reduced infectivity [2], a lower replicative capacity [3], and an increased susceptibility to antibody-mediated neutralization [4]. During the course of HIV-2 infection, CD4 declines slowly and the clinically latent phase can last for decades [5]. Still, infection with HIV-2 can lead to acquired immune deficiency syndrome (AIDS) [6] and effective antiretroviral treatments are crucial for preventing disease progression.

Possible treatments for individuals infected with HIV-2 are limited because many antiretrovirals are less effective inhibitors of HIV-2 than of HIV-1 [7–9]. HIV-2 is intrinsically resistant to non-nucleoside reverse transcriptase inhibitors [10, 11] and to the fusion inhibitor enfuvirtide [7, 12]. Additionally, from the class of protease inhibitors, only saquinavir, lopinavir, and daruinavir are effective against HIV-2 [9]. Selecting an appropriate treatment regimen can be further exacerbated by the rapid development of HIV-2 drug resistance [9, 13, 14]. Maraviroc, a CCR5 coreceptor antagonist, poses a new treatment option for individuals infected with HIV-2 [15–18]. The drug prevents viral cell entry by obstructing the CCR5 coreceptor and should be administered only to patients infected with an R5-tropic virus to ensure treatment efficacy and to prevent a switch to viral usage of the CXCR4 coreceptor. Therefore, determining viral coreceptor usage is crucial before initiating treatment with coreceptor antagonists such as maraviroc [16]. Moreover, the identification of HIV-2 coreceptor usage can be useful for staging disease progression: CXCR4-using viruses, which are less susceptible to antibody neutralization than R5-tropic strains [19], are associated with low CD4+ T cell counts and progressed disease [20, 21].

Although some HIV-2 strains have been shown to infect cells without use of the CD4 receptor in vitro [1, 22, 23], HIV-2 enters cells in vivo by first binding to the CD4 receptor and then interacting with a coreceptor belonging to the family of chemokine receptors [24]. Similarly to HIV-1, CCR5 and CXCR4 are the major coreceptors that are used by HIV-2 in vivo [25, 26]. The variable loop 3 (V3) of the viral surface glycoprotein (known as gp125 or gp105) is crucial for coreceptor binding. Specific substitutions in the V3 loop are particularly indicative of X4-capability [27–30] and often bring forth an increased V3 net charge [21, 29, 31, 32].

Three viral variants can be delineated according to the coreceptor that is used during cell entry. R5-tropic viruses can use only the CCR5 coreceptor, X4-tropic viruses can use the CXCR4 coreceptor, and dual-tropic viruses can use both CCR5 and CXCR4. Patients harboring R5- and X4-tropic viruses simultaneously have *mixed infections*. Since mixed infections usually cannot be distinguished from infections with dual-tropic variants, the term *dual/mixed* (D/M) is used to denote patients with a dual infection or a dual-tropic virus. To simplify the terminology, we define a virus/viral population as *R5* if it can use only CCR5, while *X4*-*capable* defines a virus/viral population that can use CXCR4 (possibly in addition to other coreceptors).

Viral coreceptor usage can be determined either phenotypically or genotypically. Phenotypic approaches often use engineered cell lines expressing only certain coreceptors on their surface such that they elicit a specific signal upon viral infection. For example, TZM-bl cells [33, 34] express firefly luciferase enzyme under the control of the HIV-1 promoter. Since TZM-bl cells express CD4, CCR5, and CXCR4, coreceptor usage can be measured by blocking one and/or both coreceptors with excessive amounts of coreceptor antagonists and evaluating the resulting luminescence [16, 35].

While phenotypic assays are accurate and engineered cell lines enable the detection of a broad range of coreceptor usage patterns, such assays are expensive, time-consuming, and their interpretation can be challenging. For example, when evaluating the results from an assay based on TZM-bl cells, the residual viral replication in the presence of the applied coreceptor antagonists needs to be interpreted. Moreover, TZM-bl cell based assays using different coreceptor antagonists (e.g. maraviroc and TAK-779 for CCR5) might not yield exactly the same results for the same isolate. Additionally, phenotypically determined coreceptor usage might not accord with in vivo coreceptor usage, because engineered cell lines exhibit larger surface densities of CD4 and HIV coreceptors than primary cells. Hence, a virus that cannot use a given coreceptor in vivo may be falsely reported to use that coreceptor if cell entry is enabled by the increased avidity of the interactions between virus and engineered cell. In contrast to HIV-1, where the enhanced sensitivity Trofile assay provides a standardized means for identifying coreceptor usage [36], there exists no standardized phenotypic assay for HIV-2. Instead, different phenotypic approaches are in use, which may lead to inconsistent results. Genotypic methods, on the other hand, are not performed in a laboratory, but are based on detecting discriminatory features in the viral genome. These approaches usually agree well with phenotypic tests [37], save time, and are much less expensive than phenotypic assays.

The first genotypic approach for the identification of HIV-2 coreceptor usage was put forth by Visseaux et al. [28]. Their study identified nine markers in the V3 loop exhibiting significant associations with coreceptor usage. Four of these markers with sensitivities greater than 70% and specificities of 100% were selected to form the major genotypic determinants of X4-capable variants: the substitutions L18X (where X is any non-L amino acid) and V19K/R, any insertion after position 24, and a V3 net charge exceeding six. The other five substitutions (S22A/F/Y, Q23R, I25L/Y, R28K, and R30K) with significant associations were termed minor markers. Their rules-based system classifies an HIV-2 strain as X4-capable if its V3 amino-acid sequence contains at least one of the four major markers and otherwise as R5. Applying this approach to an independent data set yielded a sensitivity of 65% and a specificity of 100% for detecting X4-capable variants.

Our study had two goals. First, we wanted to provide a data-driven, genotypic tool for predicting whether an HIV-2 V3 amino-acid sequence originates from an R5 or an X4-capable variant. More specifically, we strove to improve on the rules-based approach to coreceptor identification introduced by Visseaux et al. [28]. Second, we wanted to investigate which V3 amino-acid mutations confer the X4-capable phenotype and determine whether amino-acid features in the V1/V2 region are also predictive of coreceptor usage.

We demonstrate that viral coreceptor usage can be accurately predicted from specific amino-acid substitutions in the HIV-2 V3 loop and provide geno2pheno[coreceptor-hiv2], a web service for HIV-2 coreceptor prediction. We were not only able to confirm previously established markers of X4-capability, but also found previously unreported V3 substitutions predictive of X4-capable viruses. Additionally, we found evidence indicating that the V1/V2 region also modulates HIV-2 coreceptor usage.

## Results

To generate statistical models capable of predicting HIV-2 coreceptor usage, we gathered a data set of 126 pairs of HIV-2 genomic amino-acid sequences and phenotypic coreceptor usage annotations (either R5 or X4-capable). Based on this data set, we trained and validated support vector machines (SVMs) with various kernel functions on the amino-acid sequences of either the V1, V2, V3, or all three regions and the corresponding coreceptor usage annotations to identify the most predictive models according to their areas under the ROC curve (AUCs). Due to its high predictive accuracy, we decided to use a linear SVM based on the V3 amino-acid sequence for all further analyses. Next, we validated an existing rules-based approach for HIV-2 coreceptor identification [28] and compared the predictive accuracy of this approach with the accuracy of SVMs.

To identify which substitutions in the V3 amino-acid sequence impart the X4-capable phenotype according to the linear SVM, we investigated the model weights and statistically tested the discriminatory strength of individual substitutions in the V3 loop. Last, we implemented the linear SVM as a web service, for which we transformed predicted X4-probabilities to false positive rates (FPRs), selected a suitable FPR threshold, and created a visualization representing the model weights associated with an input sequence. To validate the implementation of the web service, we evaluated the predictive accuracy of geno2pheno[coreceptor-hiv2] on an independent set of nine new HIV-2 isolates with phenotypically determined coreceptor usage, which were not previously used for training the model.

### Model selection and validation of SVMs

To predict HIV-2 coreceptor usage, we trained SVMs on data involving several regions of the HIV-2 genome. We decided to train SVMs on the V1, V2, and V3 loops as those regions are known to impact HIV-2 coreceptor usage most [27–30, 38]. We also trained an SVM on a combination of all three variable regions. To estimate the predictive performance of SVMs on unseen data, we performed 10 runs of tenfold cross validation (CV) on the complete data set of 126 samples. Having partitioned the data set into 10 disjoint folds, the *i*-th ($i \in \left\{ {1, 2, \ldots , 10} \right\}$) round of CV entails training a model using the samples contained in all folds except for the *i*-th fold and then validating the model on the *i*-th fold. Linear models based on the V1 and V2 loops (N = 62) achieved AUCs of 0.84 and 0.65, respectively. SVMs trained on V3 amino-acid sequences (N = 126) achieved similarly high accuracies for all kernel functions considered with the exception of the SVMs based on the edit kernel, which had distinctly smaller AUCs (see Table 1). The best-performing SVM that was trained on the V3 loop outperformed the models based on the V1/V2 regions (AUC of 0.95).

**Table 1:** Classifier AUCs per run of cross validation

CV Run	RBF (σ = 0.001)	Linear	Polynomial (degree = 2)	Edit Kernel (γ = 0.005, PAM70)
1	0.9475	0.9459	0.941	0.8629
2	0.9509	0.9506	0.9452	0.851
3	0.9504	0.9579	0.9444	0.8655
4	0.9449	0.947	0.9379	0.8634
5	0.9472	0.9467	0.9413	0.8744
6	0.9467	0.9467	0.9457	0.8689
7	0.9532	0.9535	0.9475	0.8377
8	0.9522	0.9532	0.9306	0.8623
9	0.9524	0.9524	0.9478	0.9012
10	0.9441	0.9431	0.9384	0.8672
μ	0.949	0.9497	0.942	0.8654
σ	0.0033	0.0045	0.0053	0.0162

The column names indicate the kernel function corresponding to each SVM and kernel parameters are indicated in brackets. Only the results for the best-performing kernel function (in terms of average AUC across all CV runs) for each set of evaluated parameters are shown. All of the classifiers performed best with a setting of ν = 0.3

We also evaluated the performance of SVMs trained on 62 samples using the amino-acid sequences of all three variable regions V1/V2/V3 and found that the best model performed worse (AUC of 0.89) than that based on the V3 loop alone. Due to the reduced predictive accuracy of models incorporating information from the V1/V2 region, we decided to use the linear ν-SVM trained on 126 V3 amino-acid sequences with the model parameter ν = 0.3 (AUC of 0.95) for geno2pheno[coreceptor-hiv2]. We refer to this SVM as *the linear SVM* in the following.

To identify the predictive performance of SVMs trained on V3 amino-acid sequences under consideration of model selection bias, we also determined their tenfold nested CV performance. In the 10 inner runs, SVMs using a linear kernel were chosen seven times and SVMs using an RBF kernel were chosen three times using their AUCs as a selection criterion. The AUC of tenfold nested CV was 0.88 (sensitivity of 76.9% and specificity of 97.3%).

### Evaluation of the rules-based approach for HIV-2 coreceptor identification

To evaluate the rules-based approach from Visseaux et al. [28] for identifying HIV-2 coreceptor usage, we determined the predictive accuracy of their approach on a subset of the complete data set called the *test data set*. The test data set was constructed to contain only those V3 sequences that had not been used for the identification of the predictive rules used in their approach. We evaluated the rules-based approach from Visseaux et al. [28] for different numbers of required major markers of X4-capability (either 1, 2, 3, or 4) on the test data set (N = 84) and found that the balanced accuracy of prediction decreased with increasing numbers of required major markers (balanced accuracies 0.89, 0.88, 0.85, and 0.81, respectively). Hence, our evaluations confirm that requiring one major marker for X4-capability is the most accurate rules-based strategy, but the presence of additional markers can corroborate a prediction (Additional file 1: Table S1).

To determine the predictive performance of individual markers of X4-capability, we applied a two-sided Fisher’s exact test on the confusion matrices resulting from applying individual rules (Additional file 1: Table S2). After correcting for multiple hypothesis testing using the Benjamini–Hochberg procedure [39] at a false discovery rate of 5%, we found that among the established discriminatory features only the substitutions R30K and I25L/Y were not significant predictors of X4-capability on the test data set at the 5% level.

### Comparison of SVMs with the rules-based approach

To compare the predictive performance of SVMs and the rules based approach [28], we validated both approaches on the test data set (N = 84). The rules-based method from Visseaux et al. requiring just a single major rule to predict X4-capability [28] achieved a sensitivity of 85.3% and a specificity of 94% (balanced accuracy 89.6%). In comparison, tenfold nested CV of SVMs performed on the test data set resulted in a sensitivity of 73.5% and a specificity of 96% (balanced accuracy 84.7%), which is not significantly different (*p* value 0.37) to the rules-based predictions according to McNemar’s test [40].

### Discriminatory features in the V3 loop

To analyze discriminatory features in the V3 loop, we created a profile alignment of the V3 amino-acid sequences in the test data set and enumerated the positions in the V3 loop according to the HIV-2 reference strain M33262 [41–43]. Many sequences from X4-capable viruses exhibited more than one major marker for X4-capability. Of the 34 X4-capable sequences in the test data set, only 5 (14.7%) samples did not have any marker, 2 (5.9%) had a single marker, 2 (5.9%) had two markers, 4 (11.8%) had three markers, and 21 (61.8%) had four markers. Interestingly, the five X4-capable sequences without any markers for CXCR4 usage (accession numbers/isolate identifiers: DQ213035 [27], GU204944 [32], consensus V3 loop from clones JX219591-JX219598, GB87 [31], 310248 [31]) could neither be identified as X4-capable by the rules-based method nor by geno2pheno[coreceptor-hiv2].

**Fig. 1:**
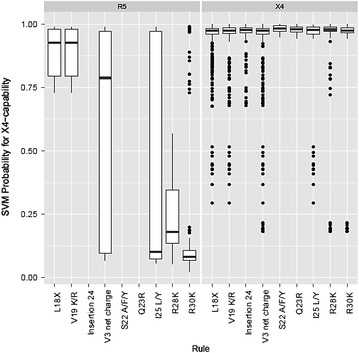
X4-probabilities predicted by geno2pheno[coreceptor-hiv2] for V3 amino-acid sequences exhibiting the established discriminatory features indicative of X4-capability listed on the x-axis. The *left-hand panel* shows the predicted X4-probabilities for sequences labeled as R5, while the *right-hand panel* shows the predicted X4-probabilities for sequences labeled as X4-capable. The *bottom line* of a *box* indicates the 1st quartile (Q1) of predicted X4-probabilities, the *bar* inside the *box* indicates the median, and the *top line* indicates the 3rd quartile (Q3). The *whiskers* extending from a *box* indicate predicted X4-probabilities that lie within 1.5× IQR (interquartile range, IQR = Q3 − Q1). Outlier values that are not within the *whisker* region are shown as *dots*. Note that some of the sequence characteristics indicated on the x-axis do not have a predicted X4-probability, because no sequences exhibiting the corresponding feature and phenotype were available

We investigated how well the linear SVM used for geno2pheno[coreceptor-hiv2] reproduces the nine previously described markers for X4-capability [28]. To this end, we visualized the predicted X4-probabilities of the linear SVM for sequences exhibiting these established discriminatory features (Fig. 1) and evaluated the SVM features contributing 75% of the total model weights (Table 2). We found that the SVM predicted high X4-probabilities for sequences from X4-capable viruses exhibiting established X4-markers, which indicates that the SVM captures the established features of X4-capable variants well. However, because some R5 sequences also exhibit markers of X4-capability (particularly L18X, V19K/R, or a V3 net charge >6), these isolates were falsely predicted to use CXCR4 with a high probability.

**Table 2:** Features in the model with the strongest impact on predicted viral coreceptor usage

Position	R5 feature	X4 feature	R5 weights	X4 weights
18	L	H, Q, F, M	0.69	−0.23, −0.15, −0.12, −0.1
Insertion after position 24	–	I, V	0.45	−0.22, −0.21
19	I	R, K, V	0.19	−0.25, −0.23, −0.19
***Insertion after position 22***	–	H, Y	0.36	−0.18, −0.18
24	P	*NA*	0.17	*NA*
23	Q	R	0.14	−0.14
***27***	Q	K	0.09	−0.12
***13***	T	R	0.11	−0.07
***26***	*NA*	N	*NA*	−0.09
***10***	A	K	0.09	−0.07
***14***	I	L	0.08	−0.08
22	S	*NA*	0.08	*NA*
***15***	A	G	0.08	−0.07
***8***	K	S	0.07	−0.07

Positions of discriminatory features that were not described previously are shown in bold italics

By analyzing the SVM model coefficients, we identified novel, discriminatory features associated with X4-capability. The substitutions 27K, 15G, and 8S were significantly predictive of X4-capability according to Fisher’s exact test at the 5% level after multiple hypothesis testing correction with the Benjamini–Hochberg procedure (Table 2).

### Predicted X4-probabilities and false positive rates

The distribution of predicted X4-probabilities resulting from applying the linear SVM on the complete data set (N = 126) using 10 runs of tenfold CV shows that V3 loops from R5- and X4-capable viruses are, for the most part, well separable (Additional file 1: Figure S1). The region of low X4-probabilities is interspersed with samples from X4-capable viruses, which indicates that the SVM falsely identifies X4-capable viruses as R5 in some cases.

To find an FPR cutoff producing a satisfactory separation of the predicted X4-probabilities from samples labeled as *R5* and *X4*-*capable*, we performed k-means clustering on the X4-probabilities after we had found $k = 2$ by applying the elbow test on the within sum of squares error [44]. From the cluster representing X4-capable viruses, we then selected the minimal predicted probability for X4-capability (53.4%) and determined the corresponding FPR (3.4%). For better memorability, we decided to set the recommended cutoff for HIV-2 coreceptor prediction to an FPR of 5%, which increases the number of false alerts only slightly (Additional file 1: Figure S2).

### The geno2pheno[coreceptor-hiv2] web service

**Fig. 2:**
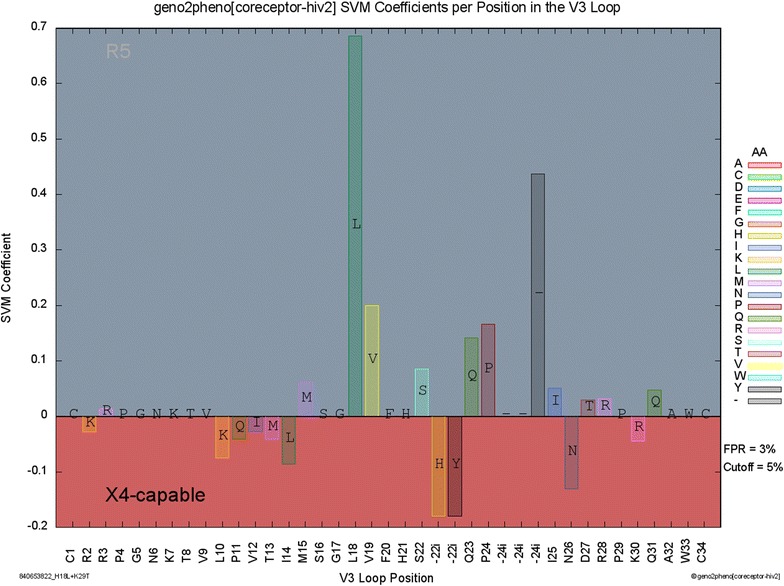
Visualization of the model coefficients for the V3 loop of the mutant ROD10 isolate (H18L + K29T). Amino acids with positive coefficients are associated with R5-tropic viruses, while negative coefficients are associated with X4-capable variants. The legend on the *right* indicates the color-coded amino acids and gives the FPR of the prediction. Because the predicted FPR is below the selected cutoff at 5%, the sequence is predicted to be X4-capable, which is indicated by the *dark color* of the X4-capable label in the *bottom left* corner. The labels of the x-axis refer to the positions and amino acids of the HIV-2 reference strain ***M33262***. Note that since the input sequence contains two insertions relative to the reference (H and Y after position 22), the 29T mutation is visualized at the x-axis tick with the D27 label

We implemented our predictive approach for the identification of HIV-2 coreceptor usage as a web service, which is available at http://coreceptor-hiv2.geno2pheno.org. After inputting one or multiple nucleotide/amino-acid sequences containing the V3 loop (at most 500) and selecting an FPR cutoff, the sequences are aligned to a profile of the V3 loop and coreceptor usage is predicted using the linear SVM. To interpret the results, the input sequences are compared to the HIV-2 reference strain M33262 [41–43]. The tool produces a PDF report showing the aligned V3 loops, provides a csv-file that tabulates the predictions for batch runs, and visualizes the model coefficients of the input sequences (Fig. 2). The visualization shows the extent to which individual amino-acid substitutions influence a prediction and enables users to gauge the evidence pointing towards a certain prediction.

### Validation of the geno2pheno[coreceptor-hiv2] web service on an independent test set

We validated the predictive performance of the geno2pheno[coreceptor-hiv2] web service on an independent test set containing nine additional V3 samples that were not contained in the data set (N = 126) that had been used to form the linear SVM of geno2pheno[coreceptor-hiv2]. Predictions from geno2pheno[coreceptor-hiv2] were compared to the phenotypically measured coreceptor usages for the nine samples, which had been determined using an assay based on TZM-bl cells. With the recommended FPR cutoff of 5%, all of the nine sequences were classified correctly (Table 3). The genotypic tool from Visseaux et al. performed slightly worse on these sequences: The R5-sequence ROD10 (H18L + H23Δ + Y24Δ) was incorrectly classified as X4-capable due to its net charge of +7 and the X4-capable sequence ROD10 (H18L + K29T) was classified incorrectly as R5, because it did not exhibit any of the major markers for X4-capability. Investigating the model coefficients of isolate ROD10 (H18L + K29T) in Fig. 2 reveals one of the strengths of geno2pheno[coreceptor-hiv2]. In contrast to rules-based approaches, geno2pheno[coreceptor-hiv2] takes into account all V3 amino acid positions, which enables the identification of coreceptor usage for viruses where a combination of substitutions enables CXCR4 usage. For example, for the ROD10 (H18L + K29T) mutant, the combination of multiple negative weights associated with the features R2K, P11K, V12K, T13M, I14L, insertions after position 22, and N26N resulted in the prediction of X4-capability, rather than fulfilling individual rules.

**Table 3:** Results from the validation of the web service on nine additional V3 sequences

Isolate	FPR	Major markers	Minor markers	Visseaux prediction	geno2pheno[coreceptor-hiv2] prediction	Phenotype
ROD10 (Wildtype)	0.01	L18X, V3 net charge >6	*NA*	X4-capable	X4-capable	X4-capable
ROD10 (K29T)	0.01	L18X	*NA*	X4-capable	X4-capable	X4-capable
ROD10 (H18L)	0.03	V3 net charge >6	*NA*	X4-capable	X4-capable	X4-capable
ROD10 (H23Δ + Y24Δ)	0.01	L18X	*NA*	X4-capable	X4-capable	X4-capable
ROD10 (H18L + K29T)	0.03	NA	*NA*	R5*	X4-capable	X4-capable
ROD10 (H18L + H23Δ + Y24Δ)	0.11	V3 net charge >6	*NA*	X4-capable*	R5	R5
ROD10 (H18L + H23Δ + Y24Δ + K29T)	0.15	NA	*NA*	R5	R5	R5
15PTHSJIG	0.36	NA	*NA*	R5	R5	R5
15PTHCEC	0.01	L18X, V19K/R, Insertion24, V3 net charge >6	Q23R, R28K	X4-capable	X4-capable	X4-capable

Incorrect predictions are marked with an asterisk. ROD10 refers to the HIV2-group A reference strain, which uses both CCR5 and CXCR4 as entry coreceptors. Mutations from the ROD10 wildtype sequence are indicated in brackets, where Δ indicates deletions

## Discussion

We were able to confirm the role of the HIV-2 V3 loop as the major determinant for the usage of the CCR5 and CXCR4 coreceptors. On the largest data set for HIV-2 coreceptor usage available to us, high predictive performances of rules-based and data-driven approaches for coreceptor identification were demonstrated. Using SVMs, we were not only able to replicate all of the established markers of X4-capable variants, but could also identify additional markers with significant predictivity that have not been described previously.

Our results substantiate three characteristics differentiating the HIV-2 and HIV-1 V3 loops with respect to coreceptor usage. While individual mutations in the HIV-2 V3 loop by themselves are highly predictive of coreceptor usage (e.g. 18X has a sensitivity of 79% and a specificity of 96%), there is no discriminatory signal in the HIV-1 V3 loop that allows for the accurate identification of coreceptor usage by itself. For example, the 11/25 rule, which classifies HIV-1 as X4-capable if its V3 loop contains positively charged amino acids at the 11th or 25th position [45], is highly specific (93%) but severely lacks sensitivity (30%) [46]. Second, while the major discriminatory markers indicating CXCR4 usage of HIV-2 (18X, 19K/R, insertions after position 24) appear at the V3 C-terminus, discriminatory features of HIV-1 coreceptor usage occur along the full extent of the V3 region. Third, while a V3 net charge exceeding six is significantly associated with the usage of CXCR4 by HIV-2 (Additional file 1: Table S2) [28], there is no significant association between the overall charge of the HIV-1 V3 loop and coreceptor usage [47], although CCR5 and CXCR4 exhibit contrasting electrostatic potential surfaces [48].

Our analysis of the predictive performance of SVMs based on various kernel functions revealed that linear kernel functions are well suited for HIV-2 coreceptor usage prediction and that kernel functions capturing higher-order interactions do not offer additional benefits in this prediction scenario. These results suggests that HIV-2 coreceptor usage is largely based on individual amino-acid mutations in the V3 loop rather than on interdependent substitutions of amino acids as in HIV-1 [49]. This finding would be supported by the hypothesized open structure of the HIV-2 V3 loop, which might reduce the role of interactions among the amino acids in the V3 loop [27]. Determining and analyzing the structure of gp125 with an intact and ordered V3 loop would be a crucial step in confirming the independence of positions by elucidating the accessibility of the V3 loop [50].

We found further evidence [38] indicating that other envelope regions besides V3 might contribute to HIV-2 coreceptor usage. First, SVMs based on the V1 and V2 regions achieved substantial predictive accuracies. Second, the V3 sequences of some X4-capable viruses did not exhibit any known features indicative of CXCR4 usage (accession numbers/isolate identifiers: DQ213035 [27], GU204944 [32], consensus V3 loop from clones JX219591-JX219598, GB87 [31], 310248 [31]) and some V3 sequences of R5-tropic isolates exhibited markers of X4-capability (Fig. 1). Third, there are several samples sharing the same V3 loop, but exhibiting discordant measurements of phenotypic coreceptor usage (Additional file 1: Table S3**)**. Note however that discordant phenotypic assignment of coreceptor use could also be the result of varying sensitivities among the different phenotypic assays (e.g. GHOST (3) cells, PBMCs with the Δ32 mutation, U87 cells) as well as experimental conditions. In case that phenotypically determined coreceptor usage is inconclusive, clarification could be obtained by genotypic approaches such as geno2pheno[coreceptor-hiv2].

R5-tropic HIV-2 viruses exhibiting X4-markers could also be explained by a switch from CXCR4 to CCR5 usage (X4-R5 reversion). X4-R5 reversions have already been reported in HIV-1-infected patients after immune reconstitution [51–54]. Because recent findings indicate that X4-capable HIV-1 viruses are less susceptible to neutralization by autologous antibodies than R5-using viruses from the same host [55], X4-R5 reversions could result from the normalization of naïve T-cell turnover following immunological recovery [56], after which the infection of naïve T-cells by X4-capable variants may not be productive enough [51]. Since X4-capable HIV-2 also seem to be less susceptible to neutralization than CCR5-using strains [19], X4-R5 reversions in HIV-2 could be explained by the same mechanism.

Besides these interpretations, discrepancies between the measured phenotypic coreceptor usage and features in the V3 amino-acid sequence could also be a by-product of the qualitative interpretation of phenotypic assays. In vivo, coreceptor usage is on a continuous scale and several, consecutive structural changes within the surface glycoprotein occurring along the viral evolutionary trajectory allow for increasingly effective coreceptor usage. However, this fact is neglected when the results of phenotypic assays are reported. Although the assays produce quantitative measurements (e.g. fluorescence, luminescence, or formation of syncytia), these measurements are typically converted to a qualitative scale for the sake of convenience regarding further analyses. Typical qualitative scales are the annotation of coreceptor usage (e.g. R5/X4-capable) or the efficiency of coreceptor usage (e.g. −/+/++/+++). For the sake of accuracy, however, it would be paramount to work on the raw, quantitative data. With quantitative measurements, it would be possible to place a virus onto the evolutionary continuum stretching from viruses using CCR5 highly efficiently to viruses capable of using CXCR4. Moreover, working on raw data from phenotypic assays would facilitate the application of established statistical techniques for the normalization of biased data arising from several experiments, which could improve the accuracy of large-scale studies on coreceptor usage considerably. Our genotypic analyses of several ROD10 mutants are a step in the right direction, because we were able to determine the impact of individual V3 substitutions on coreceptor usage quantitatively through the genotypic prediction of FPRs (Table 3).

To shed more light on the emergence of V3 amino-acid sequences with discordant phenotypic measurements, three aspects should be investigated. First, the agreement between different phenotypic assays should be validated or, even better, a standardized phenotypic assay should be developed. Second, further research investigating the intra-host evolution of HIV-2 with respect to coreceptor usage and its impact on viral fitness seems necessary to determine whether X4-R5 reversions do occur. Third and most importantly, it should be investigated whether amino acid substitutions in the V1/V2 region can impart the X4-phenotype independently of substitutions in the V3, a question for whose resolution more data is required [27].

In the following, we discuss the benefits of using geno2pheno[coreceptor-hiv2] for HIV-2 coreceptor identification. We could show that that geno2pheno[coreceptor-hiv2] outperformed the rules-based approach by Visseaux et al. [28] on an independent test set of nine V3 sequences (Table 3). Furthermore, the predictive performance of geno2pheno[coreceptor-hiv2] is at least as high as the predictive performance of geno2pheno[coreceptor] for HIV-1, whose established cutoffs (EU: 10%/20%, UK: 5.75%, Germany/Austria: 5–15%) exceed the optimized 5% cutoff that is employed by geno2pheno[coreceptor-hiv2] [57–59].

Since geno2pheno[coreceptor-hiv2] is based on an SVM, it considers all positions in the V3 loop when predicting coreceptor usage. Rules-based systems, on the other hand, use only a preselected set of discriminatory features from the V3 loop to identify coreceptor usage. This gives geno2pheno[coreceptor-hiv2] an edge over rules-based systems when coreceptor usage can only be discerned by considering combinations of multiple substitutions that together confer the X4-phenotype (Fig. 2).

The predictions by geno2pheno[coreceptor-hiv2] are not only accurate, but also interpretable. The web service visualizes the model coefficients of an input sequence to provide users a comprehensive view of the impact of individual positions on HIV-2 coreceptor usage. Additionally, geno2pheno[coreceptor-hiv2] outputs FPRs, which provide a measure of predictive confidence. Moreover, users are free to select the tradeoff between sensitivity and specificity by adjusting the cutoff for the FPR. For example, higher sensitivities (at the cost of more false alarms) can be obtained by increasing the FPR cutoff (e.g. from 5 to 20%).

## Conclusions

geno2pheno[coreceptor-hiv2] is a highly accurate and interpretable online tool for the genotypic identification of HIV-2 coreceptor usage. Using our method, we were able to obtain a better understanding of the V3 amino-acid substitutions required for the usage of the CXCR4 coreceptor and to learn more about the impact of the V1 and V2 loops on HIV-2 coreceptor usage. geno2pheno[coreceptor-hiv2] can support the clinical management of HIV-2 infection because the tool can aid physicians in taking treatment decisions and enables researchers to undertake large-scale epidemiological studies on HIV-2 coreceptor usage.

## Methods

### Supervised learning with SVMs for HIV-2 coreceptor usage prediction

Our genotypic approach to coreceptor identification is based on supervised statistical learning, more specifically, on classification. Classification requires two types of data. The first type of data is a numeric input matrix $X \in {\mathbb{R}}^{N \times p}$, where *N* gives the number of observations and *p* gives the number of features. Due to the established association between the V3 loop and HIV-2 coreceptor usage [17, 27, 29, 30], we used the amino acids of the V3 loop as features (N = 126). The input matrix was constructed such that each row $x_{i}$ contains the aligned, binary-encoded V3 amino-acid sequence of sample $\varvec{i}$. The amino-acid sequences of the V1 and V2 loops were also considered as features (N = 62), but only investigated briefly due to lacking data and smaller predictive power of the V1/V2 region.

The second type of data required for binary classification is a vector of outcomes $Y \in {\mathbb{Z}}^{N}$, whose entries $y_{i}$ contain the numeric representation of the phenotypically determined coreceptor usage of sample *i*, which is also called its label. We set $y_{i} = - 1$ for sequences labeled as *X4*-*capable* and $y_{i} = 1$ for sequences labeled as *R5*.

Because SVMs [60] based on the amino-acid sequence of the V3 region have already been used successfully for identifying the coreceptor usage of HIV-1 [61], we also decided to use SVMs. In our setting, SVMs find a vector of coefficients *α* and an intercept *β*_0_ that define a hyperplane maximizing the margin between observations from the two classes, *X4*-*capable* and *R5*. Predictions are generated by computing the decision function $f\left( {x_{i} } \right) = \sum\nolimits_{j = 1}^{N} {\alpha_{j} } y_{j} K\left( {x_{j} , x_{i} } \right) + \beta_{0}$, where $K\left( {x_{i} , x_{j} } \right)$ is a kernel function representing the similarity of two V3 loops $x_{i}$ and $x_{j}$ in Hilbert space [62]. We used LIBSVM to determine the optimal hyperplane and transform decision values to the probability that a V3 originates from an X4-capable sequence [63, 64].

### Data collection and sample labeling

The majority of the data were retrieved from the Los Alamos National Laboratory HIV database by gathering all available HIV-2 V3 sequences with annotations of phenotypic coreceptor usage [28, 32, 65–72]. Further data points were obtained from the literature [29–31] and complemented by our own phenotypic measurements, which were performed as described in the sections following Section *Cells, plasmids, and coreceptor antagonists*.

To differentiate sequences from R5-tropic strains from sequences of viruses that can use CXCR4, each observation was labeled either as *R5* or *X4*-*capable*. Isolates for which CXCR4 usage was reported (X4-tropic or D/M) were annotated as *X4*-*capable* and isolates for which only the usage of the CCR5 coreceptor was reported were annotated as *R5*. All of the isolates capable of using coreceptors other than CCR5 or CXCR4 were also able to use the CXCR4 coreceptor and therefore labeled as *X4*-*capable*.

Next, to obtain a representative training data set for statistical learning, the initial data set of 314 genotype-phenotype pairs was filtered to remove duplicate V3 sequences. During duplicate removal, we found multiple sequences with discordant annotations of coreceptor usage (i.e. sequences sharing the same V3 amino-acid sequence but having different phenotypic measurements). For each set of discordant sequences sharing the same V3 loop, we considered two possibilities: either to include one of the discordant V3 sequences into the data set or to exclude all of the sequences (Additional file 1: Table S3). In the following, we discuss each decision in detail.

Each of the samples sharing the same V3 amino-acid sequence as DQ870430 [28, 30, 32, 65–67] and NARI-12 [28, 30, 31, 65] was phenotyped as X4-capable variant only once, while a decidedly larger number of identical V3 sequences was phenotyped as R5 (21 and 5 sequences, respectively). Hence, we regarded the X4-capable measurements as outliers and the respective sequences were included with the *R5* label. The sequence with the accession GU204945 [32] was identified as X4-capable once and as R5 thrice. Hence, due to lacking evidence of actual coreceptor usage, this sequence was removed from the data set.

For the V3 sequence with the identifier 310248, usage of CCR5 and CXCR4 was reported in one study each. The sequence had been identified in the X4-capable isolate 310248 [31], but also in an R5 isolate (JN230759/isolate 29) with the same V3 sequence except for an R/K ambiguity at position 27 [28]. Interestingly, the R5 isolate showed a marginal signal for the CXCR4 coreceptor, which was discarded because the signal was <5% of the signal for CCR5 usage. Further evidence pointing towards the usage CXCR4 was presented by Owen et al. [31], who reported a minor induction of syncytia for their isolate. Additionally, applying a CXCR4 antagonist to cells lacking the CCR5 coreceptor revealed a reduction in infectivity between 40 and 90% for this strain [31], which suggests that the isolate actually seems to use CXCR4. Therefore, we included this sequence as *X4*-*capable* in our data set.

After duplicate removal and handling of sequences with discordant annotations, 126 genotype-phenotype pairs remained of which 74 (58.7%) were labeled as *R5* and 52 (51.3%) as *X4*-*capable* (Additional file 1: Table S4).The samples in the data set originate from diverse regions. In total, 87 (69%) samples were collected in Europe, of which 42 (48.3%) come from France, 33 (37.9%) from Portugal, and 12 (13.8%) from Sweden. All of the 10 (10.3%) Asian samples originate from India. Of the 24 (19%) West African samples, 15 (60%) were collected in Guinea-Bissau, 5 (20.8%) in Ivory Coast, 2 (8.3%) in Gambia, and 2 (8.3%) in Senegal.

Most isolates in the data set (84.9%) had been genotyped as HIV-2 group A. Only a minority of samples (13.5%) had been identified as group B and the remaining samples (1.6%) either had been identified as group D or had not been genotyped. The group distribution of the samples in our data set reflects the global distribution of HIV-2 groups: Groups A and B are the most prevalent genotypes and the majority of infections are caused by group A strains [42, 73, 74].

### Sequence alignment

To align the V3 sequences in the data set, we modified the Smith–Waterman algorithm for pairwise alignments [75] to perform profile alignments in order to capture the diversity of the HIV-2 V3 region. In contrast to pairwise alignments, profile alignments compare the input sequence not with a single reference sequence, but with a profile corresponding to the expected amino-acid frequencies for every position in a genomic region. We retrieved all available amino-acid sequences of the HIV-2 envelope region from the LANL HIV database and selected the V3 region through pattern matching. If a sequence exhibited the highly conserved V3 start motif (CKRP or CRRP) and the end motif (QAWC), the corresponding subsequence was selected. In cases where either only the start or end motif could be found, a search for the substring of the missing motif was conducted and the corresponding subsequence was selected if a substring of the missing motif could be found.

The extracted 1979 V3 amino-acid sequences were aligned with ClustalW version 2.1 (using the accurate switch and default parameters) [76], which is an established tool that is sufficiently accurate for identifying an overall amino acid profile of the V3 loop. We then computed the frequency of each amino acid for every alignment position to obtain a profile of the V3 loop. The profile alignment of the V3 amino-acid sequences was performed by computing the alignment scores under consideration of both, the frequency of amino acid substitutions given by the alignment profile and an amino acid substitution matrix [77].

### Sequence encoding

Let *AA* be the set of 20 amino acids augmented with the gap character “-”. To obtain the input matrix *X*, each aligned V3 amino-acid sequence *s*_*i*_ with $|s_{i} | = 39 \,\,\forall i$ was encoded as a feature vector *x*_*i*_ with 21 * 39 = 819 dimensions. Let $x_{i,j} \left[ c \right]$ denote whether the character *c* ∈ *AA* appears at position *j* in the V3 loop of observation $\varvec{i}$. To deal with ambiguous positions, we disambiguate IUPAC ambiguity codes and define *s*_*i*,*j*_ as the set of unambiguous amino acids occurring at position *j* in the *i*-th input sequence. For each position *j* in an aligned sequence *s*_*i*_, we uniformly distribute the weight among all observed amino acids and set the value of non-observed amino acids to 0:

$$x_{i,j} \left[ c \right] = \frac{1}{{{\mid }s_{i,j} {\mid }}}\quad \forall c \in s_{i,j}$$

$$x_{i, j} \left[ c \right] = 0\quad \forall c \notin s_{i,j}$$

Note that *x*_*i*,*j*_[*c*] = 1 for unambiguous positions with *s*_*i*,*j*_ = {*c*} and ${\mid }s_{i,j} {\mid } = 1$.

### Model selection and validation

Based on the input matrix *X* containing the 126 aligned and encoded V3 amino-acid sequences as well as the vector of outcomes $\varvec{Y}$ denoting phenotypic coreceptor usage, we trained several SVMs to identify which SVM performs best in terms of the AUC of the receiver operating characteristic [78]. The SVM parameter ν was varied in a range from 0.1 to 0.4 (higher values were not considered due to infeasible optimization problems) and different kernel functions (linear, radial basis function, polynomial, and edit kernel [79]) were used to form predictions.

To evaluate the performance of the SVMs, we conducted 10 runs of tenfold CV [80]. Additionally, to determine the expected performance of our approach taking into account the model selection procedure, we performed tenfold nested CV. In nested CV, two interlaced runs of CV were performed. In the inner CV run, we computed the AUCs resulting from the predictions of each model and selected the model and kernel parameters maximizing the AUC. In the outer CV run, we trained a model with the selected parameters on the inner CV training data and predicted the outcomes of samples contained in an independent fold. After all outer fold predictions had been computed, the overall model performance was determined.

To compare the performance of the rules-based approach from Visseaux et al. [28] with our method, we set up a test data set (N = 84), whose observations where not used to identify discriminatory features by Visseaux et al. This test set was formed to determine the prediction performance of their model on independent data. We evaluated whether there exists a significant difference between the rules-based approach and our method by applying McNemar’s test.

### McNemar’s test

McNemar’s test [40] is based on the values contained in a 2 × 2 confusion matrix and can be used to determine whether two classifiers perform differently. The test can be applied on paired dichotomous variables that are mutually exclusive and identifies if there exists a difference in the distribution of the marginal frequencies of each outcome. In our case, we applied the test to the predicted and phenotypically determined coreceptor usages (*R5/X4*-capable). To compare the performance of SVMs for coreceptor prediction with the rules-based approach from Visseaux et al. [28], we computed the number of samples that were correctly or incorrectly predicted by each method and constructed a 2 × 2 contingency table. The null hypothesis assumes that both approaches have the same ratio of incorrect predictions. Let *p* indicate the probability of a certain outcome. Given the entries in Additional file 1: Table S5, the underlying assumption is that *p*_*a*_ + *p*_*b*_ = *p*_*a*_ + *p*_*c*_ and *p*_*c*_ + *p*_*d*_ = *p*_*b*_ + *p*_*d*_. Hence, the null hypothesis is that $H_{0} : p_{b} = p_{c}$ and, alternatively, $H_{1} : p_{b} \ne p_{c}$.

The test statistic, ${\rm X}^{2} = \frac{{\left( {b - c} \right)^{2} }}{b + c},$ can be rejected when X^2^ is sufficiently large, that is, indicates a significant difference between the predictive performance of both approaches.

### Transformation of decision values to FPRs

We used SVMs that transform decision values to probabilities that indicate whether a V3 loop originates from an X4-capable virus (X4-probabilities) [64]. Although these probabilities give a measure of confidence, they does not afford insights into the accuracy of predictions, which is crucial for clinical applications, however. Since FPRs provide a useful measure for the confidence of a prediction and because they are an established measure for the quantification of HIV-1 coreceptor usage [61], we transformed the predicted X4-probabilities to FPRs. Here, the FPR indicates the estimated rate at which an R5-tropic virus would be falsely predicted as X4-capable when using a given X4-probability as a cutoff for the two classes.

To transform X4-probabilities to FPRs, we constructed a mapping from predicted X4-probabilities to FPRs during the training stage. Each predicted X4-probability was used as a cutoff for classifying samples once: All samples with X4-probabilities below the cutoff were assigned *R5* and all samples with X4-probabilities greater or equal to the cutoff were assigned *X4*-*capable*. This cutoff-dependent class assignment in combination with the phenotypic labels for each observation yielded a 2 × 2 contingency table indicating false positives (FP) and true negatives (TN), from which we could compute the FPR as

$$FPR = \frac{FP}{FP + TN}$$

which results from applying every predicted X4-probability as a cutoff once. Using this transformation, low FPRs indicate confident predictions of X4-capable variants, while high FPRs designate R5-tropic viruses.

### Determining the impact of amino acids in the V3 loop on HIV-2 coreceptor usage

LIBSVM outputs a weight vector $\alpha^{*} \in {\mathbb{R}}^{n}$. Its entries $\alpha_{i}^{*} = \hat{\alpha }_{i} y_{i}$ indicate the estimated weight $\hat{\alpha }_{i}$ of each support vector $x_{i}^{*}$ scaled by the corresponding outcome *y*_*i*_. The coefficients $\beta \in {\mathbb{R}}^{p}$, which reflect the impact of individual amino acids in the V3 loop on coreceptor usage, can be determined by $\beta = \alpha^{{*^{T} }} X^{*}$. Hence, given a new input sequence, $x_{i } \in {\mathbb{R}}^{p}$, we can find its amino-acid specific weights *b*(*i*) as the element-wise vector product of the coefficients and the encoded input features such that $b\left( i \right) = x_{i} *\beta$, which can be visualized in terms of a bar plot indicating the role of individual amino acids for HIV-2 coreceptor usage.

### Modified feature encoding used by geno2pheno[coreceptor-hiv2]

To predict the label of a new input sequence, its V3 is modified in two ways in order to improve predictive performance. The first modification concerns gaps in the sequence and the second relates to ambiguous positions.

Errors during sequencing or problems with the alignment can lead to the introduction of gaps in the V3 loop, which have no functional meaning and can bias predictions. Therefore, our approach detects gaps that are not functionally relevant and are likely to represent artifacts in the following way. Let $\beta_{j} \left( c \right)$ be the coefficient that corresponds to character $c$ at sequence position $j$ and let $\upvarepsilon = 0.01$.

For every position $j$ with $c = -$, we consider the model weight associated with the gap, $\beta_{j} \left( c \right)$. If $\left| {\beta_{j} \left( c \right)} \right| < \varepsilon$, the gap does not affect HIV-2 coreceptor usage according to the model and it can be replaced with the encoded consensus amino acid $a$ from position $i$ contained in the V3 alignment profile by setting *x*_*j*_[*c*] = *a* before predicting coreceptor usage for the input sequence. Otherwise, if |*β*_*j*_(*c*)| ≥ *ɛ*, no modification is necessary.

Ambiguous positions in Sanger sequencing of viral populations indicate the presence of multiple viral variants within the same host. These variants might use different coreceptors for cell entry and a single position might indicate amino acids representative of both, R5 and X4-capable viruses. To be more sensitive towards X4-capable variants, every ambiguous position in an input sequence is replaced by the disambiguated amino acids that are most strongly associated with X4-capability. Note that, since the labels for training the SVM were encoded by −1 for *X4*-*capable* and 1 for *R5*, positive coefficients designate features associated with *R5* and negative coefficients designate features associated with *X4*-*capable*.

For every ambiguous sequence position $j$ with observed amino acids *s*_*j*_, we set $s_{j} = \arg \min_{{c \in s_{j} }} \beta_{j} \left( c \right)$ in order to construct a non-ambiguous sequence that is more predictive of X4-capability. The fact that this worst-case scenario sequence might not exist in vivo when a sequence exhibits multiple ambiguous positions is only a minor concern. This is due to the following reason. Assume that a viral population consists of an R5- and an X4-capable quasispecies, which means that the prediction should be *X4*-*capable*. In this case, every ambiguous position should contain an amino acid representing the X4-capable variant such that for every ambiguous position *j* we have *β*_*j*_(*c*) ≤ 0 for all amino acids *c* occurring at the ambiguous position. Selecting the observed amino acid whose weight contributes most strongly to X4-capability means choosing the character *c* obtaining the most negative weight *β*_*j*_(*c*). Consequently, the decision value of observation *x*, *f*(*x*), enhances the prediction of X4-capable variants by reducing the decision value. The same logic can be applied to two distinct X4-capable variants. Assume now that there exist two variants that use only the CCR5-coreceptor. In this case, the prediction should be *R5* and the weights of ambiguous positions should be positive, because no amino acids associated with X4-capability are observable. Hence, the worst-case choice results in min *β*_*j*_(*c*) ≥ 0 for all characters *c* at every ambiguous position *j*, which does not enhance the prediction of *X4*-*capable* and thus does not influence the likelihood of a correct prediction of *R5* when the decision boundary is set to 0. Even for decision boundaries at values above zero, selecting the worst-case amino acid would only have a marginal effect on the prediction in the described scenario, because of the larger number and greater impact of non-ambiguous positions with positive weights.

### Cells, plasmids, and coreceptor antagonists

HEK293T cells were purchased from American Type Culture Collection (Rockville, MD). The following reagents were provided by the AIDS Research and Reference Reagent Program, National Institutes of Health: TZM-bl cells [33, 34, 81–83], TAK-779 [84, 85], and bicyclam JM-2987, a hydrobromide salt of AMD-3100 [86–88]. The wild-type pROD10 plasmid was a gift from Keith Peden [89]. HEK293T and TZM-bl cells were cultured in complete growth medium consisting of Dulbecco’s modified eagle medium (DMEM) supplemented with 10% of fetal bovine serum, 100 U/ml of penicillin–streptomycin, 2 mM of l-glutamine, 1 mM sodium pyruvate, and 1× of MEM non-essential amino acids (Gibco/Invitrogen, USA). All cell cultures were maintained at 37 °C in 5% of CO_2_.

### Virus isolates

Two new primary isolates, 15PTHSJIG and 15PTHCEC, were obtained from HIV-2-infected Portuguese patients by cocultivation with peripheral blood mononuclear cells from seronegative subjects, as described previously [90]. In addition, six new HIV-2ROD10 mutants were analyzed that contained the following mutations in the V3 loop: H18L, H23Δ + Y24Δ, K29T, H18L + H23Δ + Y24Δ, H18L + K29T, and H18L + H23Δ + Y24Δ + K29T [91]. HIV-2 ROD10 mutants were obtained by transient transfection of HEK293T cells. Transfections were performed with 10 μg of DNA in a 100 mm tissue culture dish, using the jetPrime transfection reagent (Polyplus) according to the instructions of the manufacturer. Cell culture supernatants were collected 48 h post-transfection, filtered, and stored at −80 °C.

The 50% tissue culture infectious dose (TCID50) of each isolate was determined in a single-round viral infectivity assay using a luciferase reporter assay with TZM-bl cells. First, 10,000 TZM-bl reporter cells were seeded in 96-well tissue culture plates and incubated overnight. On the next day, the growth medium was removed and replaced by 200 μl of fresh growth medium supplemented with 19.7 μg/ml of DEAE-dextran. A total of 100 μl of virus supernatant was added to the first well, from which serial threefold dilutions were prepared in the next wells. The assay was performed in quadruplets. After 48 h, luciferase expression was quantified by measuring luminescence with the Pierce Firefly Luciferase Glow Assay Kit (Thermo Fisher, USA) and the Infinite M200 luminometer (TECAN), according to manufacturer’s instructions. Control wells containing only target cells and growth medium were used to measure background luminescence. The TCID50 was calculated using the statistical method of Reed and Muench [92].

### Phenotypic determination of coreceptor usage

CCR5 and CXCR4 coreceptor usage was determined in a single-round viral infectivity assay with TZM-bl cells [16, 35]. First, 10,000 TZM-bl reporter cells were seeded in 96-well tissue culture plates and incubated overnight. On the next day, the growth medium was removed and the cells were incubated for 1 h (at 37 °C in 5% CO_2_) with growth medium either in the presence or in the absence of excessive amounts of the CCR5 antagonist TAK-779 (10 μM) and/or of the CXCR4 antagonist AMD3100 (1.2 μM). A fixed amount of virus supernatant, corresponding to 200 TCID50 was added to each well and cells were cultured with a total volume of up to 200 μl of growth medium in the presence of 19.7 μg/ml of DEAE-dextran. After 48 h, luciferase expression was quantified by measuring luminescence with the Pierce Firefly Luciferase Glow Assay Kit (Thermo Fisher, USA) and the Infinite M200 luminometer (TECAN), according to manufacturer’s instructions. Control wells containing only target cells and medium were used to measure background luminescence. A viral population was classified as R5-tropic when viral infectivity was inhibited in the presence of TAK-779 but unaltered in the presence of AMD3100, and, as X4-tropic when infectivity was inhibited in the presence of AMD3100 but unaltered in the presence of TAK-779. When infectivity was completely inhibited only by the simultaneous presence of TAK-779 and AMD3100, the virus population was classified as dual/mixed (D/M) for viral isolates or as R5/X4 tropic for ROD10 mutants.

## Additional file

**Additional file 1: Figure S1.** Distribution of X4-probabilities predicted by geno2pheno[coreceptor-hiv2]. Blue bars indicate sequences labeled as R5, while red bars indicate sequences labeled as X4-capable. **Figure S2.** Estimated TPRs versus FPRs for predictions from geno2pheno[coreceptor-hiv2]. Each dot indicates a prediction of HIV-2 coreceptor usage and the color of the dot indicates the corresponding phenotypic coreceptor usage (blue: R5, red: X4-capable). **Table S1.** Predictive performance of the rules-based approach from Visseaux et al. on the test set. **Table S2.** Predictive performance of individual rules identified by Visseaux et al. ordered by decreasing balanced accuracy as determined on the test set. **Table S3.** Overview of observations with identical V3 loops, but discordant annotation of phenotypic coreceptor usage. **Table S4.** Distribution of class labels and HIV-2 groups in the data set. **Table S5.** Structure of the 2x2 contingency table required for McNemar’s test.

## Abbreviations

SVM: support vector machine
R5: HIV-2 using the CCR5 coreceptor
X4-capable: HIV-2 capable of using the CXCR4 coreceptor
AUC: area under the ROC curve
HIV-2: human immunodeficiency virus type 2
V1/V2/V3: variable loops 1/2/3
AIDS: acquired immunodeficiency syndrome
CV: cross validation
TCID50: 50% tissue culture infectious dose
FP: false positive
TP: true positive
FPR: false positive rate
D/M: dual/mixed
