# Supplementary material for: A genotypic method for determining HIV-2 coreceptor usage enables epidemiological studies and clinical decision support
Source: Retrovirology. 2016 Dec 20;13:85. doi: 10.1186/s12977-016-0320-7 (PMC5168878; doi:10.1186/s12977-016-0320-7)
Supplement: Supplementary file 1 — Additional file 1: Figure S1. Distribution of X4-probabilities predicted by geno2pheno[coreceptor-hiv2]. Blue bars indicate sequences labeled as R5, while red bars indicate sequences labeled as X4-capable. Figure S2. Estimated TPRs versus FPRs for predictions from geno2pheno[coreceptor-hiv2]. Each dot indicates a prediction of HIV-2 coreceptor usage and the color of the dot indicates the corresponding phenotypic coreceptor usage (blue: R5, red: X4-capable). Table S1. Predictive performance of the rules-based approach from Visseaux et al. on the test set. Table S2. Predictive performance of individual rules identified by Visseaux et al. ordered by decreasing balanced accuracy as determined on the test set. Table S3. Overview of observations with identical V3 loops, but discordant annotation of phenotypic coreceptor usage. Table S4. Distribution of class labels and HIV-2 groups in the data set. Table S5. Structure of the 2x2 contingency table required for McNemar’s test. [file 12977_2016_320_MOESM1_ESM.docx]

## Supplementary Information

**Suppementary Figure 1: Distribution of X4-probabilities predicted by geno2pheno[coreceptor-hiv2].** Blue bars indicate sequences labeled as R5, while red bars indicate sequences labeled as X4-capable.

**Supplementary Figure 2: Estimated TPRs versus FPRs for predictions from geno2pheno[coreceptor-hiv2].** Each dot indicates a prediction of HIV-2 coreceptor usage and the color of the dot indicates the corresponding phenotypic coreceptor usage (blue: R5, red: X4-capable).

**Supplementary Table 1:** **Predictive performance of the rules-based approach from Visseaux et al. on the test set**.

| **Number of Rules** | **Sensitivity** | **Specificity** | **Balanced Accuracy** |
| --- | --- | --- | --- |
| 1 | 0.85 | 0.94 | 0.89 |
| 2 | 0.79 | 0.96 | 0.88 |
| 3 | 0.74 | 0.96 | 0.85 |
| 4 | 0.62 | 1 | 0.81 |

The column *Number of Rules* refers to the number of major rules (Visseaux et al.) in the V3 that were required for predicting X4-capability.

**Supplementary Table 2: Predictive performance of individual rules identified by Visseaux et al. ordered by decreasing balanced accuracy as determined on the test set.**

| **Rule** | **Sensitivity** | **Specificity** | **Balanced Accuracy** | **P-value** |
| --- | --- | --- | --- | --- |
| **L18X** | 0.79 | 0.96 | 0.88 | ${2.3\cdot10}^{-13}$ (*) |
| **Insertion after position 24** | 0.74 | 1 | 0.87 | ${3.4\cdot10}^{-14}$(*) |
| **Net charge > +6** | 0.77 | 0.96 | 0.86 | ${6.8\cdot10}^{-11}$ (*) |
| **V19K/R** | 0.74 | 0.96 | 0.85 | ${8.7\cdot10}^{-12}$ (*) |
| R28K | 0.5 | 0.96 | 0.73 | ${8.9\cdot10}^{-7}$ (*) |
| Q23R | 0.29 | 1 | 0.65 | ${4.7\cdot10}^{-5}$ (*) |
| R30K | 0.47 | 0.7 | 0.57 | 0.17 |
| S22A/F/Y | 0.15 | 1 | 0.59 | 0.009 (*) |
| I25L/Y | 0.08 | 0.97 | 0.53 | 0.47 |

The major discriminatory features are highlighted in bold. P-values were computed using a two-sided Fisher’s exact test. P-values that were significant at the 5% level after correcting for multiple hypothesis testing using the Benjamini-Hochberg procedure are indicated by an asterisk (*).

Supplementary Table 3: Overview of observations with identical V3 loops, but discordant annotation of phenotypic coreceptor usage.

| **Identifier of X4-capable Isolate** | **No. of R5 isolates** | **No. of X4-capable isolates** | **V3 loop of the X4-capable sequence** | **Decision** |
| --- | --- | --- | --- | --- |
| DQ870430 | 21 | 1 | CKRPGNKTVVPITLMSGLVFHSQPINKRPRQAWC | R5 |
| NARI-12 | 5 | 1 | CKRPGNKTVLPITLMSGLVFHSQPINTRPRQAWC | R5 |
| GU204945 | 3 | 1 | CKRPGNKTVRPITLLSGRRFHSQVYTVNPKQAWC | Exclude |
| 310248 | 1 | 1 | CRRPGNKTVVPITLMSGLVFHSQPINKRPRQAWC | X4-capable |

Supplementary Table 4: Distribution of class labels and HIV-2 groups in the data set.

| Class | Group | Frequency |
| --- | --- | --- |
| R5 | A | 61 (48.4%) |
| X4-capable | A | 46 (36.5%) |
| R5 | B | 12 (9.5%) |
| X4-capable | B | 5 (3.9%) |
| X4-capable | D | 1 (0.08%) |
| R5 | Unknown | 1 (0.08%) |

**Supplementary Table 5: Structure of the 2x2 contingency table required for McNemar’s test.**

|  | Rules-based Correct | Rules-based Incorrect | Row total |
| --- | --- | --- | --- |
| SVM Correct | a | b | a+b |
| SVM Incorrect | c | d | c+d |
| Column total | a+c | b+d | n |
